# Parathyroid Cyst and Micropapillary Thyroid Carcinoma: A Case Study of Unexpected Concurrent Endocrine Pathologies

**DOI:** 10.7759/cureus.67208

**Published:** 2024-08-19

**Authors:** Tammy Luan, Madison Guido, Elizabeth Whittington, Deanna Jantsch, Adam D Kurtin

**Affiliations:** 1 Department of Surgery, University of Miami JFK Hospital, Atlantis, USA; 2 Department of Surgery, University of Miami Miller School of Medicine, Miami, USA; 3 Department of Pathology and Laboratory, HCA Florida St. Lucie Hospital, Port St. Lucie, USA; 4 Department of Surgery, HCA Florida St. Lucie Hospital, Port St. Lucie, USA

**Keywords:** parathyroid adenoma, thyroidectomy, parathyroidectomy, parathyroid tumor, acute hypercalcemia, primary hyperparathyroidism, hypercalcemic primary hyperparathyroidism, micropapillary thyroid carcinoma, papillary thyroid carcinoma, parathyroid cyst

## Abstract

Parathyroid cysts (PCs) are rare tumors that predominantly affect middle-aged women and are characterized by symptoms of hyperparathyroidism, including fatigue, bone pain, and gastrointestinal issues. Management typically involves surgical resection due to their potential to cause profound hyperparathyroidism and hypercalcemia. PCs occasionally coexist with thyroid malignancies, including micropapillary thyroid carcinoma (MPTC), although the pathophysiological link between PCs and thyroid cancers remains unclear. We present a case of a 62-year-old woman with symptomatic hypercalcemia and a large cystic mass adjacent to the left inferior thyroid pole, initially suspected to be a PC. Preoperative imaging, including an ultrasound (US) and sestamibi scan, guided surgical intervention, resulting in en bloc resection of the PC along with a left hemithyroidectomy. Pathological examination confirmed the presence of both a PC and MPTC, raising the question as to whether there is a possible correlation between primary hyperparathyroidism (PHPT) and thyroid malignancy. This report aims to highlight the current PC management protocol, underscore the importance of thorough diagnostic evaluation and surgical strategies in addressing concurrent parathyroid and thyroid pathologies, and explore potential pathophysiological connections between these conditions.

## Introduction

Parathyroid cysts (PCs) are rare lesions, representing less than 0.5% of all parathyroid tumors [1]. The exact etiology of PCs is unclear. However, some theories hypothesize that they may be derived from embryological remnants of the third or fourth branchial clefts or cystic degeneration of preexisting adenomas. PCs are most common in women ages 30 to 50 and have been found to coexist with other conditions such as pheochromocytoma and multiple endocrine neoplasia type I [1-2].

PCs are either functional or nonfunctional. Functional PCs require the presence of both hyperparathyroidism and hypercalcemia, with calcium levels usually exceeding 13 mg/dL [2-3]. Functional PCs account for 10% of all PCs and represent the underlying cause of primary hyperparathyroidism (PHPT) in only 1-2% of individuals diagnosed with this disease [2,4]. Some individuals with functioning PCs may remain asymptomatic. In contrast, others have symptoms of hypercalcemia, including fatigue, muscle weakness, abdominal pain, decreased appetite, constipation, polydipsia, polyurea, bone pain, renal stones, or depression [1]. In addition, depending on the size and location of the PC, there may also be a palpable mass on the physical exam, as well as the presence of compressive symptoms such as dysphagia, dysphonia, or dyspnea [2].

PCs can be initially misdiagnosed as parathyroid carcinoma upon imaging with an ultrasound (US) or sestamibi scan due to their similar clinical presentations and associated symptoms [4]. However, the concomitance of a PC and a parathyroid or thyroid malignancy is infrequent. Despite being uncommon, there are a few case reports that detail coexisting PCs and non-medullary thyroid carcinomas (NMTCs). Although, it is still unclear whether there is any shared underlying pathogenesis [5-6]. While the management of PCs already involves parathyroidectomy, it is important to consider that the presence of any co-occurring parathyroid or thyroid malignancy may warrant a more conservative surgical approach or additional medical management with radiation or chemotherapy [1-2]. This report details the successful management of a woman with a coexisting PC and micropapillary thyroid carcinoma (MPTC) through parathyroidectomy and left hemithyroidectomy, highlighting diagnostic and treatment strategies for these concurrent conditions and exploring potential pathophysiological connections.

## Case presentation

A 62-year-old female with a past medical history of anxiety presented to the emergency room with complaints of weakness, decreased oral intake, constipation, and a headache. She also expressed difficulty sleeping at night due to experiencing bone pain. The patient denied any previous history of renal disease or kidney stones. Her physical exam was normal. There were no palpable masses in the neck or any apparent thyroid enlargement. 

**Figure 1:**
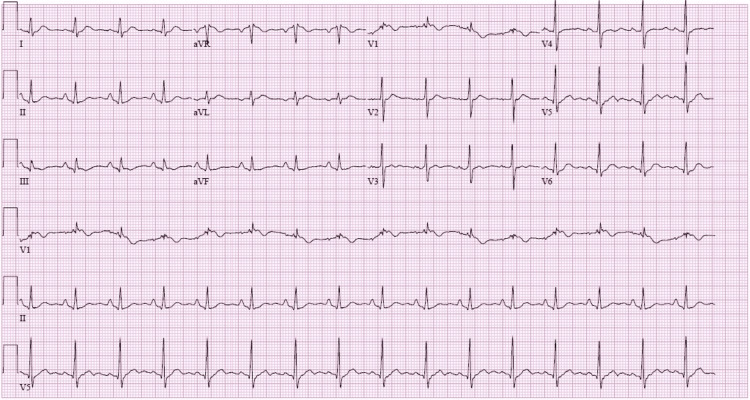
EKG with nonspecific repolarization changes in leads II, III, and aVF and J-point depression in leads V4, V5, and V6 EKG: Electrocardiogram

Initial laboratory results were significant for hypokalemia (3.3 mmol/L), severe hypercalcemia (19.1 mg/dL), elevated parathyroid hormone (PTH) (661.5 pg/mL), increased blood urea nitrogen and creatinine (25 mg/dL and 1.40 mg/dL), and a low thyroid stimulating hormone (TSH) (0.322 µU/mL) as shown in Table 1. Electrocardiogram (EKG) showed nonspecific repolarization changes in the inferior leads II, III, and aVF and J-point depression in leads V4, V5, and V6. There was no evidence of QT shortening or flattening of T waves (Figure 1).

**Table 1:** Initial laboratory results Na: Sodium; K: Potassium; Cl: Chloride; BUN: Blood urea nitrogen; Cr: Creatinine; eGFR: Estimated glomerular filtration rate; Ca: Calcium; AST: Aspartate transaminase; ALT: Alanine transaminase; PTH: Parathyroid hormon; TSH: Thyroid stimulating hormone

Parameter	Level	Reference Range
Serum Na, mEq/L	138	135-145
Serum K, mEq/L	3.3	3.5-5.1
Serum Cl, mEq/L	100	96-106
Serum bicarbonate, mEq/L	30	22-32
BUN, mg/dL	25	7-20
Serum Cr, mg/dL	1.4	0.6-1.2
eGFR, mL/min/1.73 m^2^	43	75-85
Serum Ca, mg/dL	19.1	8.5-10.2
Adjusted serum Ca, mg/dL	18.4	8.5-10.2
Total serum protein, g/dL	9.1	6.3-7.9
Serum albumin, g/dL	4.9	3.5-5
Serum AST, U/L	32	8-48
Serum ALT, U/L	27	7-55
Alkaline phosphatase, U/L	70	40-129
Intact PTH, pg/mL	661.5	15-65
TSH, µU/mL	0.322	0.5-5

**Figure 2:**
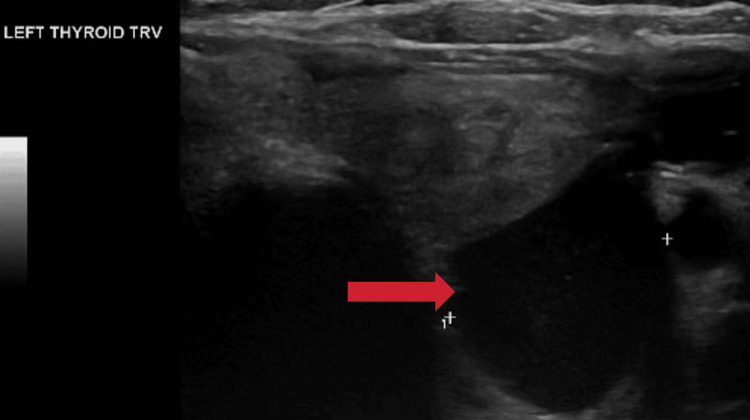
Transverse thyroid US showing a 2.4 x 2.0 x 4.4 cm hypoechoic cystic mass located posterior to the left inferior thyroid pole as indicated by the red arrow US: Ultrasound

The patient was immediately started on intravenous normal saline and zoledronic acid for treatment of severe hypercalcemia due to suspected PHPT. Preoperative imaging studies included US that demonstrated a large hypoechoic and cystic mass measuring 2.4 x 2.0 x 4.4 cm, located posterior to the left inferior pole of the thyroid (Figure 2).

**Figure 3:**
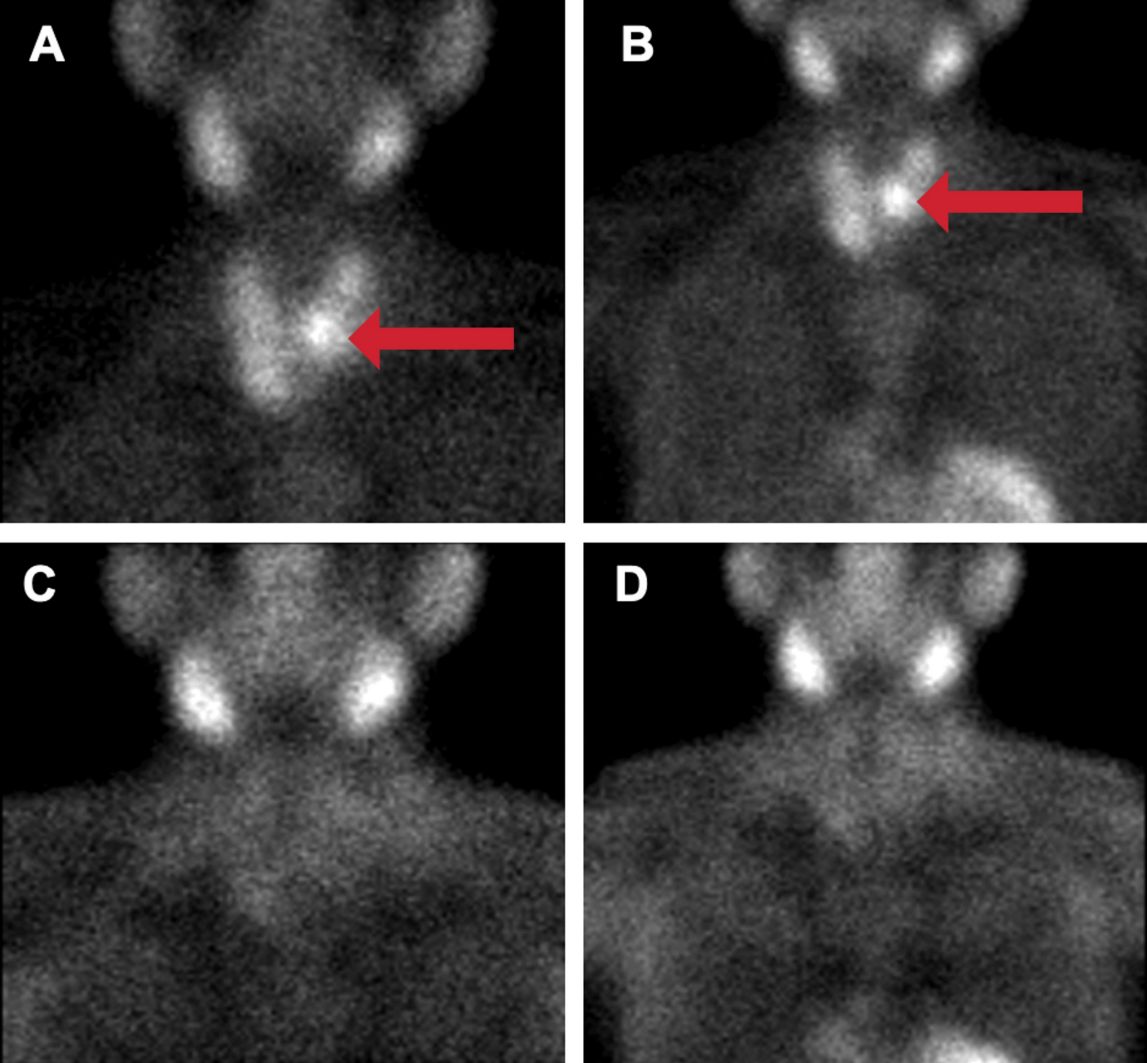
Sestamibi scan of the anterior neck and chest (A) Immediate image of the anterior neck showing increased uptake in the left inferior parathyroid gland as indicated by the red arrow. (B) Immediate image of the anterior chest showing increased uptake in the left inferior parathyroid gland as indicated by the red arrow. (C) Delayed image of the anterior neck with washout of all activity. (D) Delayed image of the anterior chest with washout of all activity.

A nuclear sestamibi scan was also completed, with initial images indicating increased uptake on the left side of the thyroid compared to the right. However, on delayed imaging, all activities were washed out and results were inconclusive (Figure 3A-3D). Despite this, the team proceeded with surgical neck exploration given the US results and profoundly elevated PTH and calcium levels. 

During surgery, a large cystic mass measuring 1.5 x 1.0 x 3.2 cm was found in the posterior aspect of the inferior pole of the left thyroid lobe. Given the patient's significant hyperparathyroidism and hypercalcemia, there was concern about a potential parathyroid carcinoma. The surgical team attempted to resect the mass en bloc to address this. However, it became evident during dissection that the mass was extensively adhered to the thyroid lobe. To ensure complete removal of the cyst and address any possible carcinoma, a left hemithyroidectomy was performed. Upon visualization of the contralateral side, two normal parathyroid glands were identified.

**Figure 4:**
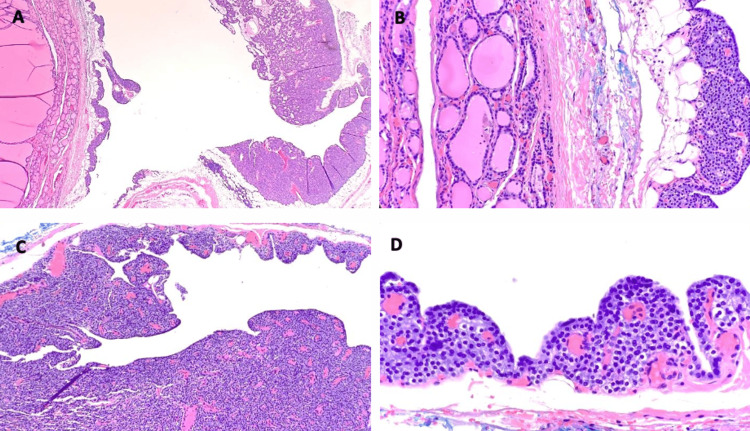
Pathology of the left thyroid lobe specimen including the cystic parathyroid mass (A) PC (right) with adjacent thyroid tissue (left) (20x, H&E). (B) PC (right) with adjacent thyroid tissue (left) (100x, H&E). (C) Hypercellular and cystic parathyroid tissue with chief cell hyperplasia, consistent with PC (100x, H&E). (D) PC comprised predominantly of chief cells (400x, H&E). PC: Parathyroid cyst; H&E: Hematoxylin and eosin

**Figure 5:**
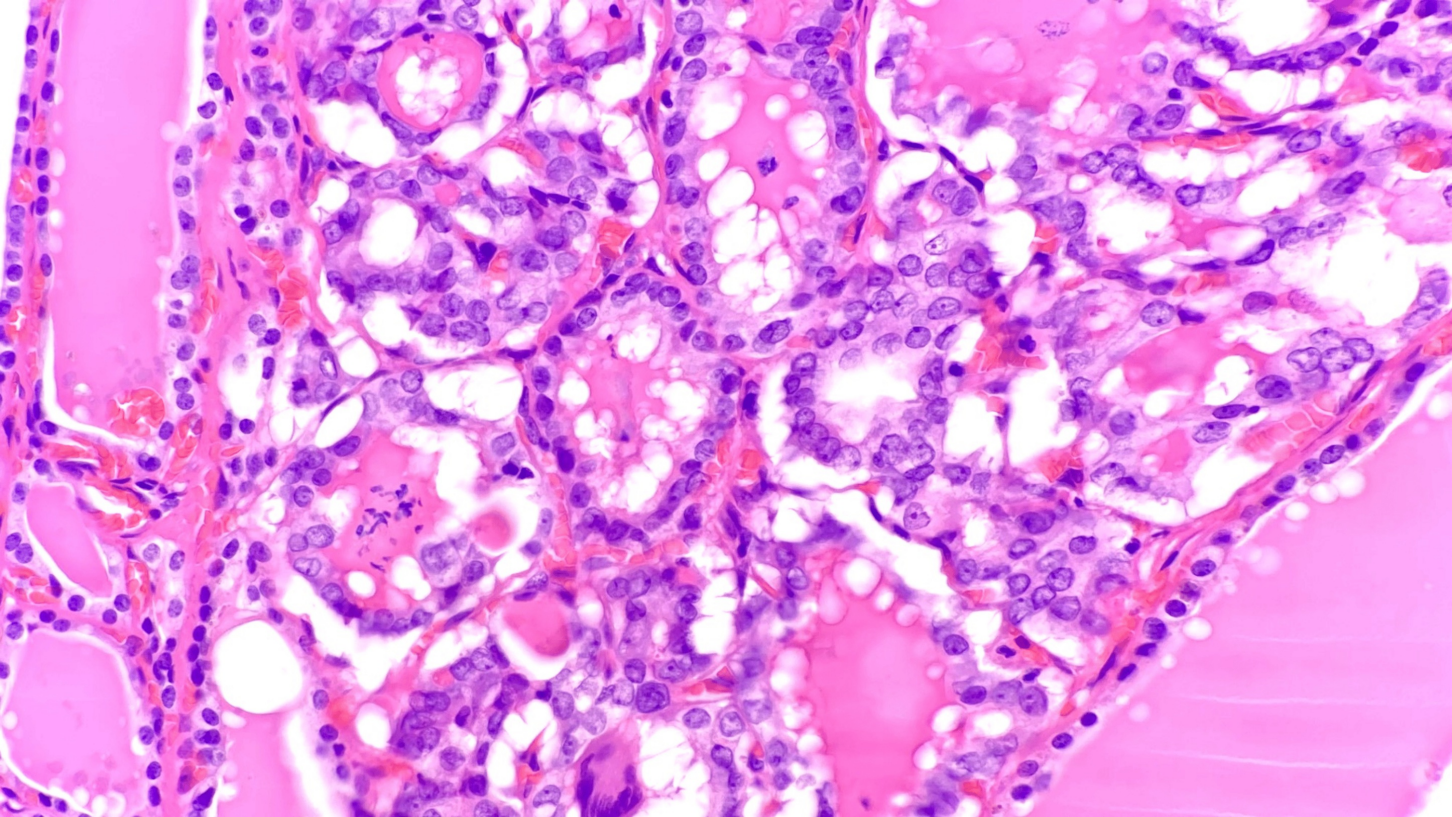
MPTC within the thyroid tissue, classic type (400x, H&E), with features including nuclear enlargement, elongation, and chromatin clearing MPTC: Micropapillary thyroid carcinoma; H&E: Hematoxylin and eosin

During intraoperative manipulation of the cystic parathyroid lesion, PTH levels rose to 1,534 pg/mL and fell to 162 pg/mL post-excision. The left thyroid lobe specimen, including the suspected PC, was sent to pathology. The final pathology resulted in a hypercellular parathyroid gland consistent with a PC (Figure 4) and a small 0.1 cm MPTC of the classic type (Figure 5). The MPTC was staged as T1M0, with less than three mitoses per mm^2^ and no evidence of lymphatic or angioinvasion.

On postoperative day one, the patient had a low calcium level of 7.9 mg/dL and complained of a mild tingling feeling in her fingertips. Otherwise, she exhibited no other signs of hypocalcemia. The patient was started on calcitriol, cholecalciferol, and calcium carbonate and then discharged on postoperative day two with an improved calcium level of 8.1 mg/dL. In the following months, the patient recovered well without complications or recurrent symptoms.

## Discussion

In this case report, we have described a rare co-occurrence of a PC and MPTC. PCs, on their own, are an infrequent disease. Epidemiologically, they are typically observed in the fourth to sixth decades of life and are more common in females [1]. The pathogenesis of PCs is not entirely clear and is likely multifactorial. Some major theories highlight the persistence of embryological remnants, coalescence of microcysts, and cystic degeneration of already existing parathyroid adenomas as potential causes [7]. Functioning PCs are usually initially recognized based on symptoms due to biochemical changes, including severe hyperparathyroidism and hypercalcemia [2].

Various imaging techniques, including US and sestamibi scans, are used to pinpoint the location of functioning PCs. However, some research suggests that the sestamibi scan's sensitivity for detecting functional PCs can be relatively low, at 29% [4]. The sestamibi scan was indeterminate in this case, as all parathyroid activity was washed out on delayed images. Despite the inconclusive overall findings of the scan, the initial images showing increased uptake on the left side of the thyroid, combined with the US that showed a left cystic mass, proved instrumental in establishing the PC's location and its relationship to the surrounding thyroid tissue prior to surgical intervention.

Certain case reports underscore the utility of fine needle aspiration (FNA) as a diagnostic modality for PCs, primarily to confirm the presence of PTH within the cystic fluid. These studies highlight how FNA may be important for surgical planning and avoiding intraoperative cyst rupture and consequent parathyromatosis [8]. Other sources discuss that it is imperative to differentiate between a benign PC and parathyroid carcinoma prior to FNA or avoid FNA altogether due to the inherent risk of tumor seeding along the needle tract in cases of carcinoma [4].

Given that we were concerned about a potential parathyroid carcinoma, we opted to proceed with surgical exploration of the neck without completing an FNA. Surgical excision is necessary to confirm a PC diagnosis via histological analysis of parathyroid tissue within the cyst wall and postoperative correction of PTH levels following cyst removal [2]. During the surgical removal of the PC from the patient, we were extremely cautious not to rupture the specimen to avoid any risk of parathyromatosis. Interestingly, during dissection, the parathyroid lesion was adhered to the left lobe of the thyroid, necessitating an en bloc resection with a left hemithyroidectomy. The final pathology resulted in a PC and MPTC, which was an unexpected finding. Due to the low-risk nature of the patient’s thyroid malignancy and the full resection of the left thyroid lobe, no further treatment was necessary for her MPTC. Nevertheless, it is important to consider the need for more conservative surgical approaches such as complete thyroidectomy or additional management with thyroid medications, radiation, or chemotherapy in patients who may have a more aggressive cancer type or advanced stage of malignancy [1-2,8].

Thyroid malignancy is the most commonly reported cancer in patients with PHPT [9]. NMTC is occasionally detected in patients with PHPT who undergo simultaneous parathyroidectomy and thyroidectomy, with reported incidences ranging from 2% to 13% [6]. Papillary thyroid carcinoma (PTC) is the most frequently occurring thyroid malignancy in conjunction with PHPT, with most cases typically involving parathyroid adenomas [9-10,11-12]. The coexistence of PCs and PTC is rare, with few case reports documenting this association [3-5].

Most published sources indicate that co-occurring PCs and thyroid malignancies are likely coincidental. Other studies hypothesize that a correlation exists specifically between the development of PCs and PTC. However, the relationship is influenced by several factors. First, they highlight how thyroid and parathyroid glands originate from the fourth and fifth pharyngeal pouches and how they share common developmental genes such as *Hoxa3*, *Pax1*, *Pax9*, *Eya1*, and *Gcm2*. This shared embryological and genetic background may predispose individuals to developing thyroid malignancies such as PTC in elevated PTH and calcium [9]. PTH has been reported to promote tumor growth and potentially initiate a DNA-altering step in the cancer development process, although, this has not been definitively established [9-10].

Additionally, it is suggested that thyroid tumor angiogenesis may be promoted by elevated levels of angiogenic growth factors, such as basic fibroblast growth factor (bFGF), influenced by the presence of PCs. Under normal conditions, bFGF is synthesized by follicular cells in the thyroid. However, its levels can surge in the presence of parathyroid adenomas or PCs, as they also contribute to its production. Individuals with parathyroid adenomas and PTC have been found to have higher levels of bFGF compared to their healthy counterparts [9]. Finally, goitrogens and neck radiation are other potential risk factors that may link the two etiologies, but these were irrelevant for the patient in this case [13]. The precise mechanism and connection between PCs and PTC or MPTC have yet to be conclusively established in the existing literature, and research is needed to obtain data that further substantiates these hypotheses.

## Conclusions

This case outlines the workup and surgical management of PCs, a rare entity that should be considered in the differential diagnosis when a patient presents with a mass in the neck or symptoms of profound hypercalcemia. The first-line treatment of PCs is highlighted as surgical resection, including en bloc resection if the mass is intimately involved with the thyroid. There are no current definitive guidelines for the management of PHPT in the context of a coexisting PC and thyroid malignancy. When considering this case, the decision to forego further surgical intervention for the patient's MPTC was based on the low-risk features of the tumor. This approach aligns with current guidelines suggesting that in cases of low-risk thyroid cancer and complete resection of the affected thyroid lobe, observation without adjuvant therapy may be appropriate. However, ongoing surveillance for both conditions is imperative, given the potential for recurrence or progression. While this report illustrates a possible relationship between PCs, PHPT, and MPTC, future studies exploring the exact molecular and genetic underpinnings linking PCs and thyroid malignancies may inform more tailored management strategies, including potential roles for medical therapies or targeted interventions, in cases where additional treatment is warranted.

## References

[1] Chaabouni MA, Achour I, Thabet W, Sellami M, Charfi S, Kallel S, Charfeddine I (2021). Parathyroid cyst: a rare entity. SAGE Open Med Case Rep.

[2] Khan A, Khan Y, Raza S, Akbar G, Khan M, Diwan N, Rizvi W (2012). Functional parathyroid cyst: a rare cause of malignant hypercalcemia with primary hyperparathyroidism-a case report and review of the literature. Case Rep Med.

[3] Ujiki MB, Nayar R, Sturgeon C, Angelos P (2007). Parathyroid cyst: often mistaken for a thyroid cyst. World J Surg.

[4] Uehara A, Suzuki T, Yamamoto Y (2020). A functional parathyroid cyst from the hemorrhagic degeneration of a parathyroid adenoma. Intern Med.

[5] Anagnostis P, Panagiotou A, Rafailidis S, Kita M (2019). Coexistence of a large functioning parathyroid cyst with papillary thyroid carcinoma: a case report and review of the literature. Case Rep Womens Health.

[6] Lehwald N, Cupisti K, Krausch M, Ahrazoglu M, Raffel A, Knoefel WT (2013). Coincidence of primary hyperparathyroidism and nonmedullary thyroid carcinoma. Horm Metab Res.

[7] Fortson JK, Patel VG, Henderson VJ (2001). Parathyroid cysts: a case report and review of the literature. Laryngoscope.

[8] Papavramidis TS, Chorti A, Pliakos I, Panidis S, Michalopoulos A (2018). Parathyroid cysts: a review of 359 patients reported in the international literature. Medicine (Baltimore).

[9] Beebeejaun M, Chinnasamy E, Wilson P, Sharma A, Beharry N, Bano G (2017). Papillary carcinoma of the thyroid in patients with primary hyperparathyroidism: is there a link. Med Hypotheses.

[10] Yazici P, Mihmanli M, Bozdag E, Aygun N, Uludag M (2015). Incidental finding of papillary thyroid carcinoma in the patients with primary hyperparathyroidism. Eurasian J Med.

[11] He Y, Liu S, Guo H, Shi B (2014). Incidental finding of papillary thyroid carcinoma with BRAFV600E mutation in a patient with coexistent primary hyperparathyroidism and Graves' hyperthyroidism. BMJ Case Rep.

[12] Mousa AH, Rahman M, Alsadeq HR, Albukhari ZZ, Ibrahim AS, Khaled I (2023). An atypical concurrent occurrence of parathyroid adenoma and micropapillary thyroid carcinoma: first case reported in Saudi Arabia. Int J Surg Case Rep.

[13] LiVolsi VA, LoGerfo P, Feind CR (1978). Coexistent parathyroid adenomas and thyroid carcinoma. Can radiation be blamed?. Arch Surg.

